# Systematic Review of Artificial Intelligence in Positive and Existential Psychiatry: Advancing Mental and Emotional Health Through Metacompetency Development

**DOI:** 10.3390/healthcare14060783

**Published:** 2026-03-19

**Authors:** Eleni Mitsea, Athanasios Drigas, Charalabos Skianis

**Affiliations:** 1 Net Media Lab & Mind & Brain R&D, Institute of Informatics & Telecommunications, National Centre of Scientific Research ‘Demokritos’ Athens, 15341 Agia Paraskevi, Greece; dr@iit.demokritos.gr; 2 Department of Information and Communication Systems Engineering, University of Aegean, 82300 Mytilene, Greece; cskianis@aegean.gr

**Keywords:** artificial intelligence, positive psychiatry, existential psychiatry, mental health, emotional health, metacompetencies

## Abstract

**Background**: Positive and existential psychiatry are approaches to mental health that emphasize the promotion of well-being, resilience, and optimal functioning alongside the conventional management of mental illness. Research suggests that the development of self-regulatory metacompetencies is associated with positive mental health and well-being outcomes. Artificial intelligence (AI) technologies are increasingly being used as assistive tools in psychiatry. However, the integration of AI in therapeutic interventions remains underexplored. **Objectives**: Thus, this systematic review aimed to synthesize evidence from randomized controlled trials evaluating whether AI-based positive and existential psychiatry interventions contribute to improvements in mental and emotional health. A second objective was to examine whether the therapeutic components and psychological processes implemented in these interventions conceptually relate to self-regulatory metacompetencies that underpin sustainable mental health and human flourishing. **Methods**: The review was conducted according to PRISMA 2020 guidelines. Only experimental studies including randomized controlled trials (RCTs) published from 2015 to 2025 were included. Twenty-four studies met the inclusion criteria. **Results**: Across interventions using conversational AI chatbots, generative AI and AI-augmented reflective systems, embodied conversational agents, social and humanoid AI robots, consistent improvements were observed in depression, anxiety, negative affect, and loneliness. The interventions enhanced various metacompetencies such as emotional regulation, emotional awareness, self-reflection, and cognitive reappraisal. **Conclusions**: The findings suggest that AI-based positive and existential psychiatry interventions can support mental and emotional health, especially when fostering key metacompetencies. Although promising, further high-quality trials are needed to clarify long-term effects. The findings of this study can contribute to the discussion about the ways AI-supported interventions may promote sustainable mental health.

## 1. Introduction

In recent years, mental health has become a major global concern, particularly due to the increasing prevalence of anxiety, depression, and emotional distress across populations [1]. Emotional health arises when an individual is capable of understanding, regulating, and expressing emotions while maintaining an optimal psychological balance and adaptive functioning in everyday life. According to the World Health Organization, mental health extends beyond the absence of illness to encompass psychological resilience, self-satisfaction, and the ability to maintain functional relationships and purpose in life [2,3]. Traditional psychiatry primarily focuses on diagnosing and treating psychopathology, yet a growing scholarly consensus suggests that psychiatry must also expand its scope to proactively foster human flourishing and psychological growth [4,5,6]. Moreover, this growing crisis underscores the need for digital, innovative, scalable, and human-centered approaches to mental health promotion and treatment [2,3].

Positive and existential psychiatry have emerged as complementary frameworks to traditional treatment models by emphasizing well-being, meaning in life, and personal growth. Existential psychiatry focuses on human experiences such as freedom, responsibility, mortality, and the search for meaning, dimensions that deeply influence mental and emotional health [7,8,9]. Confronting existential concerns can promote authentic living and emotional resilience, while neglecting them may exacerbate anxiety and depression [10,11]. Positive psychiatry integrates evidence-based interventions such as gratitude training, mindfulness, and resilience-building to enhance well-being and quality of life [12,13,14]. Such interventions aim not only to reduce symptoms but also to strengthen individuals’ capacities for self-reflection and emotional regulation, which are conceptualized as higher-order mental and emotional metacompetencies that support sustainable psychological functioning [15,16].

Metacompetencies refer to higher-order cognitive, emotional, and motivational capacities that individuals consciously and strategically use to regulate thoughts, emotions, and behaviors in adaptive ways [16,17]. Metacompetencies include, among others, self-awareness, emotional awareness, emotional regulation, and self-motivation, which are considered requirements for resilience, recovery, and long-term mental health outcomes [18,19]. Metacompetency-based interventions help individuals to develop self-regulatory and reflective abilities that guarantee psychological balance [20,21]. Studies have revealed that metacompetency training has been associated with reduced depression, anxiety, and cognitive distortions, while fostering greater autonomy and life satisfaction [22,23,24]. In the context of this review, metacompetencies are operationally defined as higher-order psychological capacities that enable individuals to consciously regulate emotions, reflect on experiences, and adaptively respond to life challenges [15,16,17,18,19]. Because these constructs are rarely measured directly in clinical trials, they are inferred through validated psychological outcomes such as emotional regulation, resilience, self-efficacy, mindfulness, and meaning in life reported across the included studies.

Parallel to these theoretical advances, artificial intelligence (AI) is transforming the landscape of mental health care by enabling personalized, accessible, and adaptive interventions [25,26]. AI-powered systems, ranging from conversational chatbots to virtual agents, are now used to deliver evidence-based therapeutic techniques such as cognitive behavioral therapy (CBT) and emotional coaching in scalable formats [27,28]. These psychological improvements are closely related to the development of metacompetencies such as emotional awareness, reflective thinking, and adaptive self-regulation, which are increasingly considered foundational for long-term mental health [15,16,17,18,19,29,30]. Moreover, AI aligns closely with the aims of positive and existential psychiatry, offering tools that can facilitate reflective dialogue, meaning-centered engagement, and sustained emotional self-regulation [31].

Despite the promising indicators, a significant knowledge gap remains regarding how AI can be effectively integrated into positive and existential psychiatry interventions to enhance mental and emotional health, taking into account the role of developing metacompetencies.

Existing research often emphasizes clinical outcomes such as symptom reduction or diagnostic precision, rather than the cultivation of long-term well-being [32,33]. It is critical to address this gap to foster a proactive, strengths-focused model in psychiatry [2,34].

Thus, this systematic review aims to synthesize evidence from randomized controlled trials evaluating whether AI-based positive and existential psychiatry interventions contribute to improvements in mental and emotional health. A second objective is to examine whether such interventions can enhance key metacompetencies that underpin sustainable mental health and human flourishing, including emotional regulation, self-regulation, and self-awareness. These metacompetencies represent higher-order psychological capacities that support adaptive functioning and long-term well-being [16,17]. Because most randomized controlled trials in this emerging field do not measure metacompetencies as primary outcomes, this review examines the extent to which the therapeutic components, psychological mechanisms, and interventions conceptually align with these metacompetencies. Specifically, this systematic review seeks to answer the following questions:

**RQ1.**  *Do AI-based interventions grounded in positive and existential psychiatry lead to improvements in mental and emotional health outcomes in randomized controlled trials?*

**RQ2.**  *How do the therapeutic components and psychological processes implemented in AI-based interventions conceptually relate to key metacompetencies that underpin sustainable mental health and human flourishing?*

This study aims to offer a systematic synthesis that examines AI-based mental health interventions through the lens of positive and existential psychiatry, rather than focusing only on symptom reduction by linking intervention outcomes to metacompetency development. It also aspires to contribute to the discussion about the ways AI-supported interventions may promote sustainable mental and emotional health. The study brings together evidence from diverse AI modalities and therapeutic techniques that are rarely discussed jointly in the existing literature. The findings can help future research to identify methodological gaps and develop more robust, ethical, and scalable AI-based mental health interventions.

This manuscript is organized as follows: Section 2 outlines the conceptual foundations of positive and existential psychiatry, metacompetency development, and the role of artificial intelligence in mental health interventions. Section 3 describes the methodology of the systematic review conducted in accordance with PRISMA guidelines. Section 4 presents the results of the included randomized controlled trials, followed by a discussion of the findings, implications, and limitations in Section 5. Finally, Section 6 summarizes the main conclusions and highlights directions for future research.

## 2. Conceptual Foundations of AI-Assisted Positive and Existential Psychiatry

### 2.1. Existential Psychiatry and Meaning in Life

Existential psychiatry emphasizes the exploration of human existence, personal meaning, and individual freedom as central components of mental and emotional health [7]. The existential perspective, drawing from existential philosophers such as Kierkegaard, Nietzsche, Heidegger, and Frankl, highlights the importance of addressing existential concerns, including mortality, isolation, and the search for meaning [8,9]. When individuals experience a “loss of meaning,” they may develop profound psychological distress, including depression, anxiety, or existential vacuum [35]. Conversely, the pursuit of meaning in life is associated with psychological well-being and resilience [36].

Existential psychiatry empowers individuals to take responsibility for their well-being, explore personal values, and construct meaning even amid suffering [11]. Individuals are encouraged to address maladaptive coping mechanisms, such as withdrawal or dependence, and cultivate adaptive strategies rooted in purpose and responsibility [37]. By acknowledging the inherent anxieties and paradoxes of human existence, existential psychiatry promotes not merely symptom relief but a transformation toward greater self-understanding and genuine living [38].

### 2.2. Positive Psychiatry and Well-Being

Positive psychiatry extends the traditional focus of psychiatry toward the enhancement of well-being, resilience, and psychosocial functioning [12,39]. Positive psychiatry employs strengths-based interventions that foster optimism, gratitude, social connectedness, and meaning in life [13,40].

Evidence indicates that positive psychiatry interventions, such as strengths-based exercises, mindfulness, and gratitude practices, contribute to lasting reductions in depressive and anxiety symptoms, while enhancing overall life satisfaction and quality of life [14]. In patients with bipolar disorder and other mood disorders, structured positive psychiatry programs have led to improved emotional regulation, resilience, and recovery outcomes [40]. It is important to highlight that positive and existential psychiatry are considered complementary approaches to traditional psychiatric methods that together foster both the alleviation of distress and the cultivation of flourishing [6].

Positive and existential psychiatry are complementary approaches that have the potential to expand mental healthcare beyond symptom reduction, focusing on meaning, well-being, and flourishing.

### 2.3. Metacompetencies in Mental Health

Metacompetencies refer to higher-order cognitive, emotional, and motivational abilities that individuals consciously and strategically employ to reflect upon, regulate, and adapt cognitive and emotional mechanisms in response to life challenges [15,16]. Metacompetencies integrate metacognition (self-reflective awareness of one’s own thoughts), emotional intelligence (capacity to recognize and regulate emotions in self and others), and self-motivation (intrinsic drive toward meaningful goals) [41,42].

Metacompetencies refer to a set of consciousness-raising capacities such as self-observation, self-regulation, and adaptation. Metacompetencies also include the ability to recognize emotions, to discern functional from dysfunctional states of mind or feelings, and consciously self-induce states of mind that awaken the positive aspects of self. The terms also indicate the individuals’ ability to be adaptive and accept changes with an open and positive attitude, directing attention to goals rather than obstacles. Metacompetencies also allow one to deliberately employ thoughts and feelings as a means to increase intrinsic motivation and improve psychological balance [15,16].

In the context of mental health interventions, several metacompetencies appear across empirical studies and therapeutic approaches. Among the most frequently addressed capacities are emotional regulation, referring to the ability to understand and manage emotional responses. Self-awareness involves reflective understanding of one’s thoughts, emotions, and behaviors. These psychological capacities represent higher-order skills that enable individuals to navigate emotional challenges and maintain psychological balance over time [15,16,17,18,19].

Metacognitive competencies, such as cognitive monitoring and self-reflection, reduce symptoms of anxiety, depression, and obsessive–compulsive disorder [22]. Emotional intelligence metacompetencies, particularly emotional awareness and regulation, improve psychosocial functioning and quality of life among individuals diagnosed with schizophrenia or bipolar disorder [18]. Similarly, self-motivation and autonomous goal-setting have been associated with better treatment adherence, resilience, and psychological well-being [19]. Thus, cultivating metacompetencies represents a shift in psychiatry, one that emphasizes psychological agency, adaptability, and self-regulation as key therapeutic goals [21].

### 2.4. Artificial Intelligence Revolution in Psychiatry

Artificial intelligence is recognized as a promising force in psychiatry [43]. AI technologies, including chatbots, adaptive learning systems, and self-management apps, are increasingly used to deliver interventions that aim to promote mental and emotional health [27,29,44]. Recent studies outline that AI-assisted therapeutic interventions can promote psychological growth, self-understanding, and emotional adaptability. Personalized feedback, interactive dialogue, and data-driven adaptation are only a few advantages of AI systems that may advance psychiatric interventions [28,31]. AI-based interventions can integrate real-time feedback and continuous interaction, supporting users in applying independent psychological techniques [30,45]. Such dynamic engagement aligns with the principles of psychiatry, which now aim not only at symptom reduction but also personal growth, meaning construction, and flourishing [34,46].

By conceptualizing AI not only as a therapeutic delivery tool but also as a facilitator for existential self-development, psychiatry can move toward a more integrative and human-centered model of digital mental health [43,47]. This perspective positions AI as a facilitator of continuous self-learning and self-reflection [16]. AI-driven interventions may hold the potential to improve psychiatric care by cultivating human potential rather than managing pathology [43,48]. From this perspective, AI technologies may serve as practical tools for delivering therapeutic strategies derived from positive and existential psychiatry.

Existential and positive psychiatry provides the foundation for a strengths-based and meaning-oriented approach to mental health. When enhanced through AI technologies, these paradigms can move psychiatry from a reactive to a proactive model, empowering individuals to develop the metacompetencies necessary for self-regulation, resilience, and growth [15,16,38]. This theoretical synthesis underpins the current systematic review, which explores how AI-assisted positive and existential psychiatric interventions cultivate mental and emotional health.

To ground the conceptual foundation of the present review, the main theoretical domains informing the manuscript are summarized in Table 1. The table outlines the key principles of positive and existential psychiatry, the role of metacompetencies in psychological functioning, and the potential contribution of artificial intelligence-assisted interventions in supporting these processes. Presenting these elements in a structured format facilitates the understanding of the conceptual relationships guiding this review and highlights the motivation of the study, namely, to examine whether AI-supported interventions grounded in these perspectives may contribute to improvements in mental and emotional health outcomes. Together, these conceptual domains form the theoretical framework guiding the present review. Specifically, positive and existential psychiatry provides the therapeutic orientation, metacompetencies represent the psychological mechanisms of change, and artificial intelligence functions as the technological delivery system enabling scalable mental health interventions.

**Figure 1:**
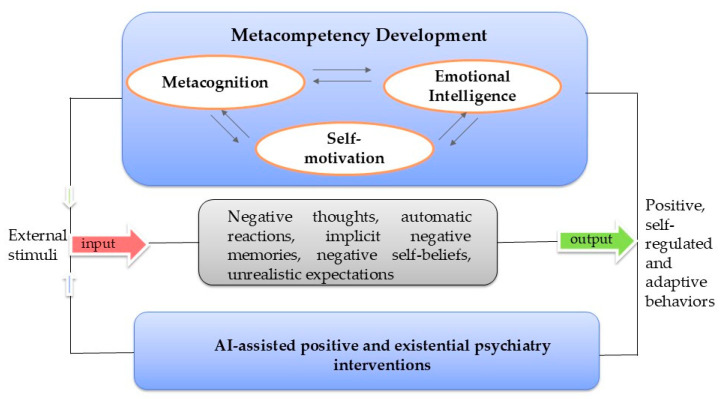
The Conceptual Model of the AI-Enhanced Healthcare Approach in Positive and Existential Psychiatry. This diagram illustrates the systemic integration of AI delivery mechanisms with positive and existential therapeutic orientations. It also highlights the central role of metacompetencies, which integrate core competencies related to emotional intelligence, motivation, and metacognition. These metacompetencies work synergistically with AI delivery mechanisms and the principles of positive and existential psychiatry to empower individuals to transform maladaptive behaviors into healthy, positive, and growth-oriented ones, ultimately driving sustainable mental health and flourishing.

A primary motivation for this review stems from the need to move beyond symptom reduction and toward a proactive healthcare approach, a conceptual model for sustainable mental health that prioritizes the development of psychological agency (Figure 1). The current review also recognizes the critical need to identify scalable, human-centered methods that address the existential and positive dimensions of human experience, which are frequently overlooked in traditional digital health interventions. Consequently, this study aims to systematically synthesize evidence from randomized controlled trials to determine how AI interventions facilitate these processes and to what extent they contribute to human flourishing.

## 3. Materials and Methods

### 3.1. Study Design

This systematic review was conducted by following the principles set out in the PRISMA 2020 statement, which provides structured guidance for planning, identifying, screening, and synthesizing research evidence. The completed PRISMA checklist is also provided as Supplementary Materials [49]. The purpose of the review was to explore whether AI-driven interventions grounded in positive psychiatry improve mental and emotional health, and whether these improvements are conceptually linked to the development of metacompetencies. A detailed protocol describing the review question, eligibility criteria, and planned procedures was registered in the Open Science Framework (https://osf.io/jvtkn/overview, accessed on 7 January 2026, doi: 10.17605/OSF.IO/4275K) [50]. The review process took place from April 2025 to October 2025 and was carried out by a team of three researchers who independently screened articles, checked eligibility, and extracted data.

### 3.2. Eligibility Criteria

In the current systematic review, participants of any age, including clinical and non-clinical populations, were included. A requirement was to collect studies delivering techniques linked to positive or existential psychiatry (i.e., meaning-making techniques). As regards the digital design of the intervention, the current study focused on AI-based tools, such as chatbots, generative AI, rule-based conversational agents, embodied/humanoid agents, or AI-guided journaling tools, designed to improve mental or emotional health by delivering positive or existential psychiatry techniques. Studies conducted before the widespread introduction of generative artificial intelligence systems were eligible for inclusion if they employed AI-driven or automated digital interventions supporting mental health outcomes. This decision was based on the recognition that current generative AI applications have evolved from earlier forms of artificial intelligence, including conversational agents, rule-based chatbots, adaptive digital platforms, and machine-learning-supported therapeutic systems. Excluding studies before 2022 would have overlooked foundational empirical evidence informing the development of present AI-assisted mental health interventions. Therefore, inclusion across the last decade was considered necessary to capture the technological and conceptual continuity underlying AI applications within positive and existential psychiatry.

Moreover, it was necessary to select studies that reported at least one validated measure related to mental or emotional health, and the measurement tools can be associated with the development of metacompetencies. As regards the study design, priority was given to experimental studies and, more specifically, to randomized controlled trials. The current review selected articles published in English in peer-reviewed journals. Another criterion was the date of publication. This study selected studies published from 2015 onwards to capture the most recent developments in AI-based interventions. Studies that employed digital design without AI components were excluded. Reviews, technical reports, and non-experimental studies were also excluded. Research protocols or design frameworks without reporting outcomes were not included. A summary of the inclusion and exclusion rules is shown in Table 2 in the main manuscript.

### 3.3. Information Sources

Searches were conducted in the following five academic databases: Web of Science, PubMed, PsycINFO, Scopus, and Google Scholar. The aforementioned academic databases were selected because they provide access to interdisciplinary research across psychiatry, digital health, and artificial intelligence. Reference lists of the included studies were also reviewed to identify additional relevant papers.

### 3.4. Search Strategy

The search strategy combined keywords and subject headings related to artificial intelligence, positive psychiatry interventions, and metacompetency development. More specifically, the following terms were prioritized: “artificial intelligence,” “AI,” “chatbot,” “conversational agent,” “virtual agent,” “embodied agent,” “robot,” “positive psychiatry,” “wellbeing,” “mindfulness,” “cognitive behavioural therapy,” and “emotional regulation.” Boolean operators were applied to ensure a broad search. The complete search strings used for each database are provided in the Supplementary Materials (Tables S1–S5). Table 3 shows the main keywords used in the search strings.

### 3.5. Selection Process

The retrieved records were imported into Mendeley, a reference-management program that automatically removed duplicate entries. Two reviewers independently screened the titles and the abstracts of the remaining records. Studies that did not meet the inclusion criteria were excluded. The full texts of the remaining studies were reviewed in detail. The reviewers independently assessed each article for methodological quality, presence of an AI component, use of positive-psychiatry techniques, and reporting of psychological and metacompetency outcomes. Each reviewer classified a study as “include”, “exclude”, or “uncertain”. Discrepancies were resolved through discussion, and when consensus could not be reached, the third reviewer provided the final decision.

### 3.6. Data Collection Process

Data extraction was conducted independently by two reviewers using a structured extraction form. For each eligible study, the reviewer recorded the following information: author(s) and publication year, study design, country, and sample characteristics, AI-based digital design, positive psychiatry techniques used, the intervention’s duration and/or number of sessions, outcome measures for mental and emotional health, and outcomes related to mental or emotional health and metacompetency development. This information was organized in a structured table (see Appendix A) to support synthesis.

### 3.7. Data Items

Data were collected on outcomes related to mental and emotional health as well as metacompetency development, because these domains aligned with the research questions. The primary mental-health outcomes included depression, anxiety, stress, mood, positive and negative affect, subjective well-being, resilience, loneliness, and emotional exhaustion. Secondary outcomes included broader indicators of emotional functioning, such as emotional stability, life satisfaction, coping skills, and perceived psychological support. As regards metacompetency development, the review extracted the measures related to metacompetencies, including self-awareness, emotional awareness, self-regulation, emotional regulationmental and emotional flexibility.

### 3.8. Reporting Bias Assessment

As regards the risk of bias assessment, the Cochrane Risk-of-Bias 2 (RoB 2) tool was selected to evaluate randomized trials (RCTs), because it is specifically designed to evaluate bias in the conduct and reporting of RCTs and aligns with the requirements of systematic reviews of interventions [51]. Moreover, RoB 2 focuses on trial-level domains that are associated with internal validity (i.e., randomization process, deviations from intended interventions, missing outcome data, measurement of the outcome, and selection of the reported result).

## 4. Results

### 4.1. Study Selection and Characteristics

Twenty-four randomized controlled trials were selected. The review included randomized controlled trials and randomized feasibility studies. The studies were conducted between 2015 and 2025 across multiple countries, including the USA, China, Germany, Poland, Japan, Turkey, Singapore, Indonesia, and Australia.

Across the included studies, several categories of AI modalities were identified, including conversational AI chatbots, generative AI–based dialogue systems, rule-based therapeutic chatbots, AI-assisted reflective writing tools, embodied conversational agents, and socially assistive robots. AI-delivered positive and existential psychiatry interventions in the included corpus integrated an array of positive-psychiatry techniques such as gratitude exercises, savoring, positive reappraisal, values clarification, behavioral activation, and self-affirmation, which were deployed either as brief prompts or as structured multi-session modules. In the included trials, these metacompetency-related constructs were typically assessed using validated psychological scales measuring domains such as emotional regulation, psychological well-being, resilience, mindfulness, and self-efficacy. These outcomes were considered indicators of the broader metacompetency framework guiding the present review. The duration of interventions varied from single-session designs to 12-week programs. Participant samples ranged from small pilot studies with 28 participants to large-scale trials involving over 600 participants, covering various populations such as adolescents, university students, and clinical or subclinical groups experiencing depression, anxiety, stress, loneliness, or burnout. The selected studies employed validated psychological scales to assess outcomes.

Table 4 provides a synthesized overview of the randomized controlled trials grouped by the primary AI modality used in the interventions. The table summarizes key methodological and intervention characteristics, including the number of studies within each AI category, typical sample sizes, intervention duration, the positive or existential psychiatry techniques employed, the metacompetencies developed, and the principal mental and emotional health outcomes reported.

**Figure 2:**
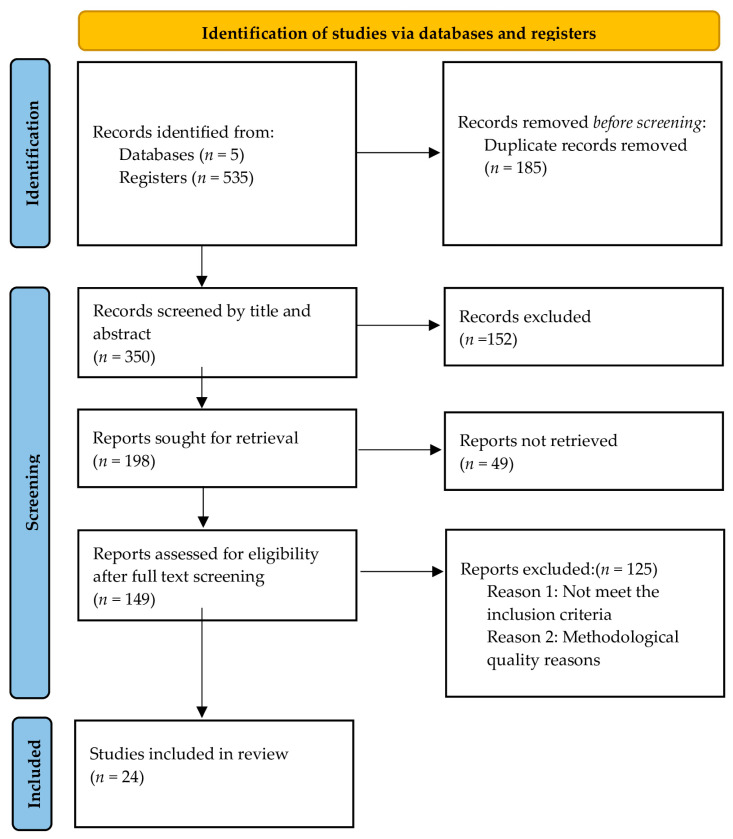
The PRISMA flow diagram.

The study selection process followed the Preferred Reporting Items for Systematic Reviews and Meta-Analyses (PRISMA) guidelines [49]. Figure 2 presents the PRISMA flow diagram summarizing the identification, screening, eligibility assessment, and final inclusion of studies in this review. The diagram illustrates the number of records retrieved from the selected databases, the removal of duplicates, the screening of titles and abstracts, and the full-text assessment of potentially relevant articles, leading to the final set of studies included in the analysis.

### 4.2. The Main Findings

#### 4.2.1. RQ1: Can AI-Based Positive and Existential Psychiatry Interventions Improve Mental and Emotional Health?

Across the full set of included studies, there is accumulating evidence that AI-based interventions grounded in positive psychiatry lead to improvements in mental and emotional health. Across the studies, AI-assisted interventions were deployed in diverse positive psychiatry contexts, integrating techniques related to the enhancement of emotional regulation, the cultivation of adaptive future orientation, training in socioemotional meta-sompetencies, and support for coping with psychological symptoms.

The evidence comes from randomized controlled trials featuring a wide range of AI modalities, including generative-AI chatbots, rule-based conversational agents, AI-augmented journaling systems, mobile cognitive-behavioural therapy chatbots, and embodied or humanoid robotic agents. Despite variation in therapeutic content and delivery format, the findings indicated that AI-mediated psychological interventions have the potential to reduce symptoms of depression, anxiety, stress, loneliness, and psychological distress, while simultaneously enhancing well-being, positive affect, resilience, and emotional stability.

The most replicated evidence concerns reductions in depressive and anxiety symptoms which appeared across various randomized trials [52,53,54,55,56,57]. One of the most robust examples comes from Heinz et al. [58], whose generative-AI mental-health chatbot delivered Socratic questioning, reflective listening, and structured emotional-regulation exercises over four weeks. Participants in the AI condition reported significant reductions in depressive and anxiety symptoms, alongside improvements in emotional awareness and self-compassion.

Another randomized controlled trial evaluated an AI intervention where participants received two weeks of access to a text-based conversational agent delivering CBT techniques (i.e., solution-focused techniques) [59]. The procedure involved the AI sending daily check-ins and providing personalized coping strategies and mindfulness exercises based on user input. The findings indicated that the group using the AI experienced statistically significant reductions in symptoms of depression and anxiety compared to the waitlist control group. It was revealed that the AI was effective in promoting users’ emotional self-regulation skills.

Similar findings emerged in Fulmer et al. [52], where an AI-based positive-psychiatry and CBT-oriented chatbot produced substantial declines in symptoms among a large sample of users (626 participants randomly assigned to chatbot or control conditions), with clear reductions in depression and anxiety and increases in emotional well-being and resilience over an eight-week intervention period

A parallel pattern can be observed in studies targeting subclinical or moderate affective symptoms. The study conducted by Karkosz et al. [53] showed that a web-based AI therapy chatbot delivering cognitive restructuring and behavioural activation over two weeks significantly reduced anxiety-related indices compared to controls. Decreases in loneliness were also noted, suggesting broader socio-emotional benefit.

Klos et al. [60] likewise reported reductions in anxiety and depression among university students following an eight-week AI-guided intervention combining relaxation, problem-solving, and emotion-focused strategies, with corresponding gains in emotional well-being and behavioural activation.

Several studies extended these findings to clinical populations. Akdogan et al. [61] found that an AI-delivered empathetic and reflective counseling intervention for young adults with anxiety and depression produced significant reductions on the Hospital Anxiety and Depression Scale (HADS), with improvements in emotional self-regulation, metacognitive awareness, and self-efficacy. Jiang et al. [62] evaluated AI-based meditation and coping-skills chatbots among adolescents with psychological disorders and observed reductions in anxiety, depression, and negative emotional states over a two-month intervention, indicating clinically meaningful symptom relief in a younger population. For cancer patients, an RCT by Akdogan et al. [61] reported that AI counseling produced lower anxiety and depression levels compared with controls, demonstrating feasibility and effectiveness in highly vulnerable groups.

He et al. [63] conducted a three-arm single-blind RCT with young adults showing depressive symptoms, comparing an AI mental health chatbot and a control group. The chatbot group engaged in daily guided conversations, focusing on cognitive restructuring, emotion regulation, and behavioral activation. Procedures included mood tracking, interactive exercises, and psychoeducation. After eight weeks, the chatbot group showed significant reductions in depressive symptoms and improvements in emotional regulation, self-reflection, and coping skills.

Beyond anxiety and depression, several studies showed compelling evidence for improvements in stress, burnout, and emotional exhaustion. Kleinau et al. [55] conducted a large-scale RCT in Malawi among 511 health workers. It was found that the AI chatbot, delivering cognitive restructuring, gratitude exercises, breathing, relaxation, and meditation, after eight weeks of intervention, decreased burnout and increased resilience. Tong et al. [64] employed an AI chatbot that provided users with psychoeducation on positive psychiatry techniques. The results indicated a reduction in depressive symptoms. However, these significant improvements were not maintained at the one-month follow-up assessment.

Sturgill et al. [44] observed that an AI-powered emotional-intelligence and mindfulness app reduced anxiety and depression in college students while improving overall emotional wellness and mood regulation over 14 weeks.

The evidence for a reduction in loneliness and improvement in social connectedness is also notable. Indrayanti et al. [57] demonstrated that an AI app-based psychological first-aid chatbot significantly reduced loneliness and improved psychological well-being after a single 30 min session, showing that even brief AI interactions can produce rapid socio-emotional benefits.

Robinson et al. [56] found that a humanoid autonomous social-robot delivering mindfulness-based well-being training increased positive feelings and self-satisfaction in a large sample, reinforcing the potential of embodied AI as an affective-support tool.

AI-based positive psychiatry interventions that employed techniques such as gratitude, savoring, and positive reappraisal provided additional evidence for improvements in mood, positive affect, and affective balance. Lee et al. [54], for instance, reported that a three-day gratitude-chatbot program increased positive affect, decreased negative emotions, and enhanced gratitude among adult users, with significant emotional-well-being gains compared to the control group.

Hung et al. [65] demonstrated similar findings in two experiments showing that a single 10 min session with a chatbot delivering gratitude and self-affirmation exercises can enhance life satisfaction and emotional regulation. Greer et al. [29], working with young adults after cancer treatment, found that a chatbot offering kindness, savoring, and reappraisal exercises can reduce anxiety and depressive symptoms after 12 sessions, emphasising the viability of AI tools for post-treatment psychological support.

Another important direction of evidence concerns the role of AI-augmented reflective tools. Zulfikar et al. [66] evaluated an AI-augmented journaling system assisting users in drawing on memories to imagine positive futures. Over two weeks, participants experienced reductions in anxiety and depression and improvements in daily affect, reflecting the therapeutic capacity of AI to scaffold reflective meaning-making and emotional insightfulness. Similar benefits emerged in Fitzpatrick et al. [27], whose fully automated CBT chatbot reduced depression and anxiety in young adults within two weeks by guiding users through reflective exercises and behavioral activation.

Interventions based on mindfulness and acceptance-based techniques also consistently improved psychological health. Anan et al. [46] implemented an AI-assisted acceptance and mindfulness program over twelve weeks. Improvements were observed in depressive and anxiety symptoms, as well as enhanced capacity for managing intense feelings. Gardiner et al. [67] found that an embodied conversational agent delivering mindfulness-based lifestyle recommendations can improve stress regulation and mood over four weeks. Increases were also observed in adaptive coping and mindfulness indices.

Across all intervention modalities, studies converge on the conclusion that AI tools combined with positive and existential techniques have the potential to improve mental and emotional health. Improvements were observed not only at symptom-level indices (depression, anxiety, panic, stress) but also in positive-affect metrics, gratitude, mood stability, resilience, and overall psychological wellness. Notably, even short AI interactions (as brief as 10–30 min) were sufficient to yield measurable improvements in emotional states, while multi-week interventions tended to produce more robust effects.

Despite these consistent benefits, several limitations should be acknowledged. Many interventions demonstrated improvements only at post-treatment or short follow-up, leaving long-term durability uncertain. Variability in outcome measures, control conditions, and intervention intensity limits comparability across studies. Attrition rates were occasionally high in longer interventions, and some outcomes relied on self-reported measures. Nonetheless, the repeated observation of significant improvements across diverse populations, settings, and intervention formats provides evidence that AI-based positive and existential psychiatry interventions are not only feasible but clinically impactful in enhancing mental and emotional health.

#### 4.2.2. RQ2: How Do the Psychological Mechanisms and Therapeutic Techniques Used in AI-Based Interventions Relate to Metacompetencies?

Across the selected studies, AI-assisted positive and existential psychiatry interventions improved a set of metacompetencies. These metacompetencies can be understood as regulatory, reflective, and adaptive capabilities that enable individuals to navigate emotional challenges, sustain adaptive functioning, and manage psychiatric symptoms [15,16]. Although the included trials did not explicitly label these constructs as “metacompetencies,” several outcome measures captured related psychological capacities, including emotional awareness, adaptive coping, self-reflection, and resilience.

A prominent metacompetency trained across multiple interventions is emotional self-regulation, which is evident in studies designed to address anxiety, depressive symptoms, and panic-related distress [58]. Similarly, the AI-assisted counseling intervention for anxiety and depression in oncology patients indicated structured support for modulating negative affect and improving affective stability during medical stressors [61]. Interventions examining AI-delivered positive behavioral techniques in panic disorder or general anxiety and depressive symptoms [28,60] imply the strengthening of emotional regulation metacompetencies by encouraging individuals in recognizing, reframing, and managing maladaptive emotional responses. Emotional self-regulation is further implicated in AI tools employing techniques for improving positive thinking, mindfulness, or emotional intelligence among college students [44]. Embodied conversational agents delivering mindfulness recommendations similarly reinforced regulatory abilities through attention training and calmness-oriented practices [67].

A widely trained metacompetency was reflective meaning-making, essential within existential psychiatry. An AI-based journaling system employed by [66] enabled participants to “draw from memories to imagine positive futures,” a process that cultivated autobiographical integration, personal meaning, and future-oriented coherence, which are considered core capacities in existential psychiatric recovery. By prompting users to reinterpret past experiences and envision emotionally sustaining trajectories, the system trained metacognitive reflection and existential orientation. Other chatbot-based interventions demonstrated that reflective training can assist individuals in adjusting to life after stressful and traumatic situations [29]. The intervention improved identity reconstruction, uncertainty tolerance, and emotional reorientation, each representing existential metacompetencies frequently emphasized in psychiatric survivorship work.

Another frequently targeted metacompetency is interpersonal-emotional attunement, visible in interventions designed to address loneliness, relational distress, or socio-emotional deficits. Indrayanti et al. [57] found significant improvements in the participants’ capacity to re-establish interpersonal connections and internalize feelings of relational safety.

Interventions deploying humanoid robots for brief well-being training [56] or embodied virtual agents supporting depression treatment [45] fostered self-regulation metacompetency. The psychological artificial intelligence system TESS, described as relieving symptoms of depression and anxiety, enhanced users’ interpersonal and flexibility metacompetencies [52].

The AI chatbot interventions involving gratitude and self-affirmation exercises [65] trained the individuals to recognize intrinsic strengths, internalize affirming self-narratives, and counteract negative self-concepts—mechanisms central to psychiatric models of identity stabilization. Gratitude-focused chatbot systems [54] also engaged capacities for self-reflection, emotional reframing, and recognition of supportive aspects of one’s environment.

Metacompetencies supporting cognitive control and symptom management also appear frequently across AI-based interventions. Cognitive-behavioral therapy chatbots that employed psychoeducation and structured cognitive reframing strategies [27] trained metacompetencies of cognitive restructuring and emotional problem-solving. Similar AI systems for panic disorder [28] and ADHD-related challenges [30] strengthened attentional control and self-control. Help4Mood, a virtual agent-based system supporting depression treatment, trained self-monitoring, behavioral adaptation, and meta-awareness of mood fluctuations [45].

In addition to psychiatric metacompetencies related to emotion and cognition, several interventions cultivated self-management and adaptive functioning, particularly in contexts where physical symptoms intersect with mental health. AI-based self-management apps [34] and AI-assisted programs for workers with chronic pain and anxiety [46] targeted metacompetencies such as behavioral regulation. Although these studies focus on somatic conditions, the capacity for sustained self-management is a recognized psychiatric metacompetency, influencing mood stability and perceived control. GymBuddy and Elomia are AI-integrated applications that similarly focused on improving self-management and daily functioning in academic and personal domains [62].

Interventions targeting loneliness [57] indicated benefits in interpersonal connectedness and social-emotional adjustment. Gratitude-based systems [54] and positive self-affirmation chatbots [65] pointed to enhanced emotional orientation, improved affective tone, and reinforcement of protective identity schemas. The Resonance system’s orientation toward imagining positive futures [66] indicated benefits associated with strengthened future orientation and existential coherence, which are widely recognized as protective factors in psychiatric recovery.

Studies implementing AI to support individuals facing medical or situational stressors—such as cancer survivors [29] or college students during the COVID-19 pandemic [44]—suggested benefits in coping metacompetencies, stress regulation, and emotional stabilization during heightened vulnerability.

### 4.3. Outcome Measure Heterogeneity and Comparative Interpretation

The included randomized controlled trials employed a wide range of psychometrically validated outcome measures to assess mental and emotional health effects [55,58,61,62]. Standardized instruments frequently used in psychiatric research, such as the Hospital Anxiety and Depression Scale (HADS) [28,29,30,31,32,33,34,35,36,37,38,39,40,41,42,43,44,45,46,47,48,49,50,51,52,53,54,55,56,57,58,59,60,61] or the Generalized Anxiety Disorder Scale (GAD-7) [58], were not consistently applied across studies. Instead, investigators selected outcome measures aligned with specific intervention targets, populations, or therapeutic frameworks. These included measures of depression [29,31,32,33,34,35,36,37,38,39,40,41,42,43,44,45,46,47,48,49,50,51,52,53,54,55,56,57,58,59,60,61,62], anxiety [29,61], stress [30,54,55,57,65], resilience [52,55], emotional regulation [44,61,63], and meaning-related functioning [54,66].

Imposing uniform measurement requirements would have resulted in the exclusion of a substantial proportion of relevant randomized trials, particularly given the methodological diversity characteristic of emerging AI-assisted mental health interventions [32,33,61]. Digital and AI-based interventions frequently aim to influence broader psychological functioning beyond symptom severity alone [15,31], leading researchers to employ diverse but clinically validated psychometric instruments [20,21,42].

To enable meaningful comparison despite measurement variability, intervention effectiveness was interpreted based on the direction and consistency of reported outcomes rather than direct numerical comparison between scales. Improvements were therefore synthesized according to whether studies reported reductions in psychological distress [58,62] or enhancements in adaptive psychological functioning within validated outcome domains [56,66]. This approach allowed the representation of the available evidence while minimizing exclusion bias and preserving the conceptual scope of the review [49]. Nevertheless, variability in outcome measures limits direct comparison of effect magnitude across studies and highlights the need for future research employing more standardized assessment frameworks alongside measures capturing higher-order psychological capacities [15,43,48].

### 4.4. Cross-Study Synthesis of Convergent Findings

Across the included randomized controlled trials, several patterns were observed regarding the impact of AI-assisted interventions grounded in positive and existential psychiatry. Most studies reported significant improvements in at least one mental or emotional health outcome, particularly reductions in depressive and anxiety symptoms and improvements in emotional regulation and psychological well-being.

Reductions in depression and anxiety were the most frequently replicated findings. Improvements in these outcomes were reported across different AI intervention formats, including conversational agents and chatbot-based cognitive behavioral interventions [27,58,63]. Similar reductions in anxiety and depressive symptoms were also observed in university students and community samples receiving AI-guided emotional regulation and problem-solving interventions [44,60]. Evidence from clinical or vulnerable populations further supported these findings. For example, chatbot interventions delivered to cancer survivors or individuals experiencing medical stressors also showed reductions in anxiety and depressive symptoms while supporting coping capacities [29,61].

Beyond symptom reduction, several studies reported improvements in psychological capacities associated with metacompetency development. Enhancements in emotional regulation, self-reflection, resilience, and coping abilities were frequently reported across interventions employing reflective dialogue, gratitude exercises, or mindfulness techniques [52,55,56]. Gratitude and affirmation-based chatbot systems similarly improved positive affect and life satisfaction [54,65]. In addition, AI-assisted journaling and future-orientation interventions contributed to improvements in reflective functioning and emotional insight [66].

Although the studies used different technological architectures and outcome measures, the direction of effects was generally similar across trials. Similar benefits were observed across rule-based chatbots, generative AI conversational agents, mindfulness applications, and AI-augmented reflective tools. These results suggest that different technological modalities may facilitate similar therapeutic processes associated with positive and existential psychiatry.

Some variability in results was nevertheless identified. Deviational factors included differences in intervention duration, participant characteristics, and levels of user engagement with the digital platforms. Short interventions sometimes produced immediate improvements but showed weaker maintenance at follow-up [64]. Differences in AI system design and therapeutic techniques may also explain variations in effect magnitude across studies. Despite these methodological differences, the overall pattern of findings indicates a consistent tendency toward improved mental and emotional health outcomes following AI-assisted interventions.

### 4.5. Risk of Bias Assessment

**Figure 3:**
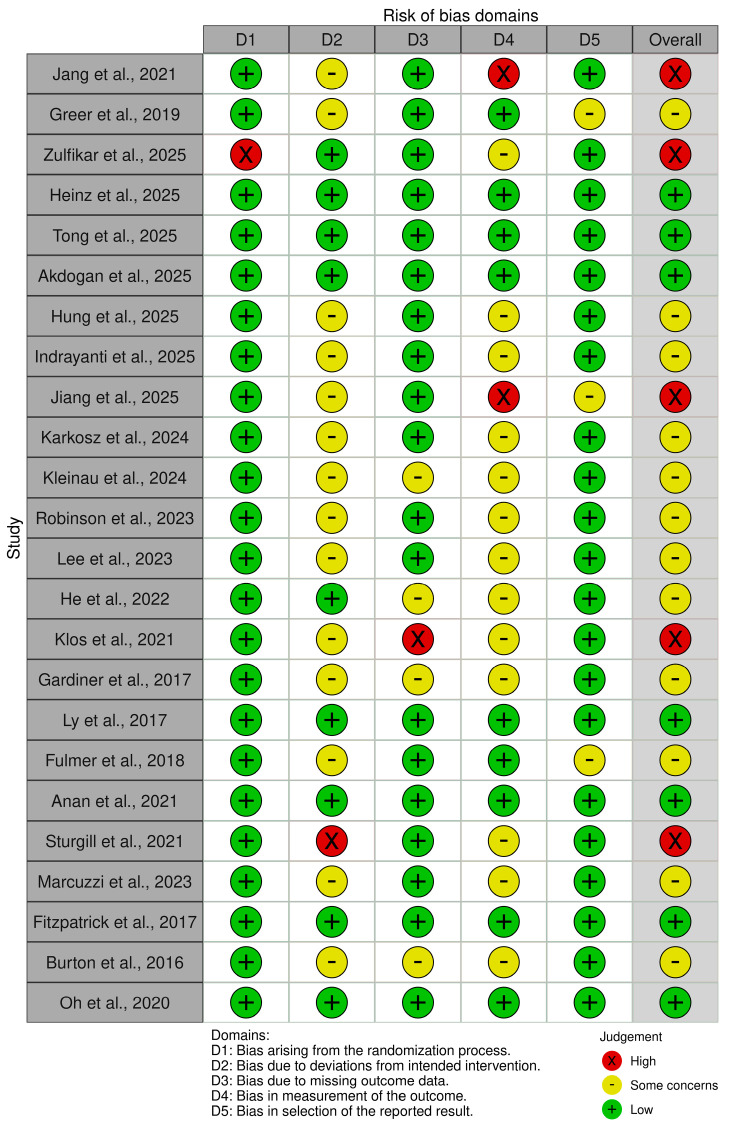
ROB-2 ”traffic light” plots visualize the risk of bias of each randomized controlled study selected in this review based on five domains of assessment [27,28,29,30,34,44,45,46,52,53,54,55,56,57,58,59,60,61,62,63,64,65,66,67].

**Figure 4:**
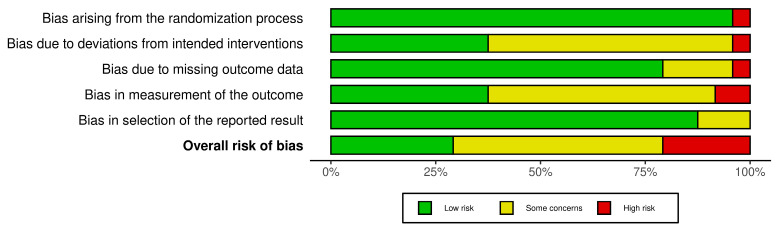
ROB-2 weighted bar plots visualize the distribution of the risk-of-bias of the selected randomized controlled trials based on five domains of assessment.

The methodological quality of the included randomized controlled trials was evaluated using the Cochrane Risk of Bias 2 (RoB 2) tool across five domains: bias arising from the randomization process (D1), bias due to deviations from intended interventions (D2), bias due to missing outcome data (D3), bias in measurement of the outcome (D4), and bias in the selection of the reported result (D5) [51]. The overall risk-of-bias judgments for each study are summarized in Figure 3 and Figure 4.

Overall, the majority of the included studies demonstrated low risk of bias or some concerns, indicating a generally acceptable methodological quality across the evidence base. Several studies were assessed as having low overall risk of bias [28,58,59,61,63,64]. These studies demonstrated low risk across most domains, reflecting appropriate randomization procedures, minimal deviations from intended interventions, limited missing outcome data, and reliable outcome measurement strategies.

Several studies were rated as having some concerns, primarily due to incomplete reporting of methodological procedures rather than clear methodological flaws [29,52,53,54,55,56,57,63,65,67]. In these cases, the primary concerns were related to limited reporting regarding adherence to intervention protocols, incomplete description of allocation concealment procedures, or insufficient clarity regarding pre-specified statistical analysis plans.

A smaller number of studies were judged to have a high risk of bias overall, mainly due to issues identified in one or more domains. For instance, the studies conducted by Jang et al. [30] and Jiang et al. [62] were rated as high risk primarily due to concerns in the measurement of outcomes and other methodological limitations. Similarly, Zulfikar et al. [66] presented a high risk associated with the randomization process, while Klos et al. [60] demonstrated a high risk related to missing outcome data. Additionally, Sturgill et al. [44] were judged as having a high risk due to deviations from intended interventions.

At the domain level, most studies demonstrated low risk of bias in the randomization process (D1), suggesting that the majority of trials used appropriate random allocation procedures. Nevertheless, a few studies, such as Zulfikar et al. [66], showed concerns related to insufficient reporting of the randomization methodology.

Regarding deviations from intended interventions (D2), most trials were judged to have either low risk or some concerns. However, one study conducted by Sturgill et al. [44] showed a high risk due to potential deviations from the planned intervention procedures.

For missing outcome data (D3), the majority of studies were assessed as having low risk of bias, indicating that attrition rates were generally acceptable and appropriately addressed in the analyses. Nevertheless, Klos et al. [60] demonstrated a high risk in this domain due to concerns related to incomplete outcome data.

In terms of measurement of outcomes (D4), most studies used validated self-report or standardized psychological assessment instruments. However, Jang et al. [30] and Jiang et al. [62] were judged to have a high risk in this domain due to potential issues related to outcome measurement procedures.

Finally, for selection of the reported results (D5), most studies were evaluated as having low risk, although several were categorized as having some concerns due to limited reporting regarding pre-specified outcome analyses or trial protocols.

Taken together, the risk-of-bias assessment indicates that the overall methodological quality of the included randomized controlled trials was generally satisfactory, with the majority of studies demonstrating low risk or only some concerns across domains. While a small number of studies presented a higher risk in specific domains, these limitations were not pervasive across the evidence base.

## 5. Discussion

This systematic review examined randomized controlled trials evaluating AI-assisted positive and existential psychiatry interventions aimed at promoting mental and emotional health through the lens of metacompetency development. Across the 24 included trials, evidence demonstrated that AI chatbots, embodied conversational agents, and generative AI systems have the potential to enhance mental and emotional health. Almost all studies reported statistically significant improvements in at least one mental health outcome, including reductions in depressive symptoms [58,63,66], anxiety [29,60], distress [64], or loneliness [57].

**Figure 5:**
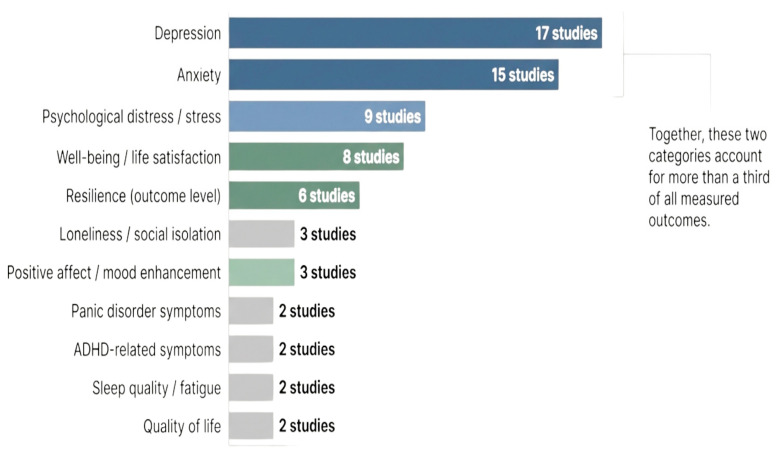
The most frequent mental and emotional health improvements.

As shown in Figure 5, depression was the most frequently evaluated outcome, reported in 17 studies [27,55,58,66]. Anxiety outcomes were assessed in 15 studies, often alongside depression [29,60,61]. Psychological distress or stress-related outcomes were reported in 9 studies, including perceived stress, burnout, and emotional exhaustion [55,64,67]. Well-being and life satisfaction outcomes were identified in 8 studies, reflecting broader positive psychiatry goals beyond symptom reduction [54,56,65]. Clinically relevant outcomes included loneliness (3 studies) [53,57] and condition-specific outcomes such as ADHD symptoms or panic disorder severity (3 studies) [28,30].

The interventions targeted a broad spectrum of positive or existential psychiatry techniques, including gratitude, savoring, mindfulness, meaning-making, self-reflection, optimism, and cognitive reframing. Mindfulness-based and emotion-focused techniques were also employed to foster present-moment awareness and non-judgmental acceptance of emotional states. These techniques helped users to identify and reframe cognitive patterns as well as encourage proactive mood-lifting activities. These psychological interventions mapped onto metacompetencies such as emotional awareness, self-regulation, cognitive reframing, self-compassion, stress management, and self-efficacy.

**Figure 6:**
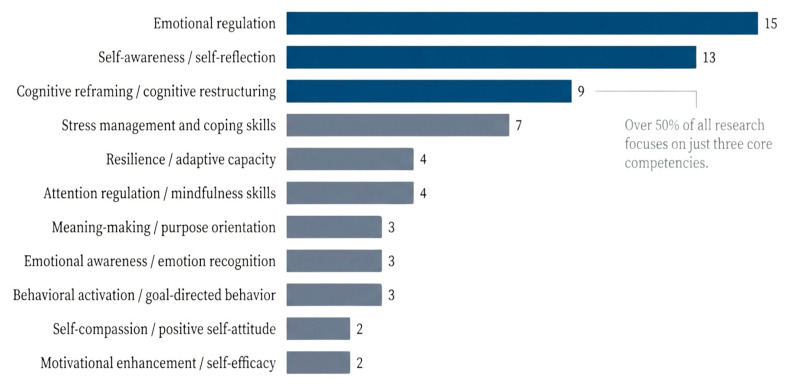
The main metacompetencies were developed after AI-assisted positive and existential psychiatry interventions.

Across trials, as shown in Figure 6, several core metacompetencies were repeatedly targeted. Emotional regulation emerged as the most frequently addressed metacompetency and was found in 15 studies, including trials using cognitive restructuring, mindfulness, gratitude, and reflective dialogue [52,55,60,61,66]. Self-reflection and self-awareness were found in 13 studies, often facilitated through journaling, Socratic questioning, or reflective chatbot dialogue [27,54,58,59]. Cognitive reframing, a metacompetency closely linked to adaptive meaning-making, was identified in 9 studies, particularly those grounded in positive behavioral activation frameworks [52,53,63]. Improved stress management was mentioned in 7 studies, often alongside relaxation, mindfulness, or solution-focused strategies [29,55,60]. Adaptation or resilience-related capacities were reported in 4 studies, mainly in larger trials emphasizing long-term psychological flexibility [52,55]. Attention regulation and mindfulness skills were identified in 4 studies, particularly those using meditation-based or embodied agent designs [30,46,56].

Socratic questioning–based chatbots improved cognitive reframing and emotional insight [53,58]. Gratitude and savoring-based AI systems enhanced positive thinking and self-awareness [54,65]. Mindfulness-oriented AI agents supported attention control and stress management [56,67].

AI tools with appropriate prompts trained individuals to observe, recognize, interpret, and regulate internal states of mind, gradually strengthening emotional and cognitive balance. The repeated structured dialogues appeared sufficient to consolidate metacompetencies such as emotional recognition [52], cognitive reappraisal [61], and problem-solving [63]. The fact that even single-session interventions [57,65] produced immediate emotional benefits highlights the potential of AI to improve mental and emotional health metacompetencies.

As regards the type of AI modality, this review concludes that different AI architectures may support different metacompetency pathways. More specifically, it was observed that generative AI chatbots [58] demonstrated reflective capabilities, enhancing emotional awareness and self-compassion. Rule-based or topic-specific chatbots produced reliable but modest improvements, particularly in short interventions [64]. Embodied conversational agents or humanoid robots [56,67] showed significant effects on mindfulness and self-satisfaction. AI CBT chatbots consistently reduced anxiety and depressive symptoms [27,28].

Positive and existential psychiatry strategies assisted by AI technologies helped participants effectively manage external triggers as a resource for initiating positive, self-regulated, and adaptive behaviors.

Across trials, AI interventions yielded results comparable to those of brief human-delivered positive or existential psychiatry programs, particularly in symptom reduction and emotional regulation [38]. AI can deliver techniques such as gratitude journaling, cognitive reframing, and mindfulness consistently and at scale, offering advantages including: 24/7 accessibility, non-judgmental interaction, high adherence with minimal cost, immediate feedback, and guided reflection.

The results of this review align with and extend earlier evidence demonstrating the effectiveness of digital positive psychiatry interventions in enhancing emotional well-being. Prior meta-analyses of digital positive psychology tools have shown small-to-moderate improvements in well-being and reductions in depressive symptoms [68,69], and the findings from AI-based systems in the present review are comparable, suggesting that conversational interactivity may amplify engagement and metacompetency acquisition. Early chatbot studies demonstrated significant reductions in depression and anxiety over two weeks [27], and similar effects were observed across recent generative AI and rule-based chatbots included in this review [58,60]. Moreover, consistent with prior work highlighting that emotionally responsive AI can enhance user self-reflection and emotional articulation [70,71], the current findings showed that metacompetencies (i.e., emotional awareness, cognitive reframing, and self-regulation) improved across a wide range of AI modalities. Studies on mindfulness apps have previously shown benefits for stress reduction and attentional control [72], and similar outcomes were replicated in AI-mediated mindfulness trials such as Robinson et al. [56] and Gardiner et al. [67]. Most importantly, the present results also converge with earlier research suggesting that digital interventions may be particularly effective for populations with subclinical symptoms or mild-to-moderate distress [73], with AI systems showing efficacy across non-clinical samples, students, and community adults. Overall, the consistency between the current findings and prior literature indicates that AI-based positive and existential psychiatry not only fits within the broader landscape of digital mental health but may offer unique advantages in personalization, scalability, and interactive skill development that go beyond earlier digital tools.

### 5.1. Motivational Factors and Psychological Suffering

A central perspective within existential psychiatry involves the simultaneous consideration of psychological suffering and motivational processes that support meaning, agency, and personal growth. Across the included studies, intervention outcomes predominantly reflected reductions in psychological suffering, commonly assessed through decreases in anxiety, depressive symptoms, stress, or emotional distress. These findings indicate the capacity of AI-assisted interventions to alleviate symptom burden.

In addition to symptom reduction, several studies reported improvements in psychological capacities associated with motivational functioning. These included enhanced emotional regulation, increased self-reflective awareness, strengthened coping abilities, resilience, and greater engagement in value-oriented or goal-directed behaviors. Although not always explicitly conceptualized as motivational constructs within primary studies, such outcomes reflect processes related to personal agency and adaptive engagement emphasized in existential and positive psychiatry.

Within the context of this review, motivational factors are therefore understood as psychological capacities that facilitate active adaptation to life challenges rather than mere reduction of distress. The coexistence of suffering reduction and motivational enhancement suggests that AI-assisted interventions may support both stabilization of mental health symptoms and development of adaptive psychological functioning. Nevertheless, given that motivational constructs were indirectly inferred from available outcome measures, these interpretations should be considered preliminary and warrant further empirical investigation using explicitly integrated assessment frameworks.

### 5.2. The Contribution of Artificial Intelligence: Opportunities and Challenges

Nevertheless, limitations relative to human clinicians remain as shown in Figure 5. Most AI systems lack deep empathic attunement, contextual nuance, and the ability to tailor complex relational interventions. The evidence also suggests that AI may be more effective for mild to moderate symptoms rather than severe psychopathology (i.e., trials involving clinical depression showed moderate but not large effects) [45,63]. As regards the limitations of the selected studies, some small RCTs showed unclear randomization procedures, blinding limitations, or insufficient reporting of missing data [59,61]. Some interventions had a duration under four weeks, and few included long-term follow-up, leaving the durability of metacompetency gains uncertain. Differences in AI sophistication were rarely controlled or systematically compared.

**Figure 7:**
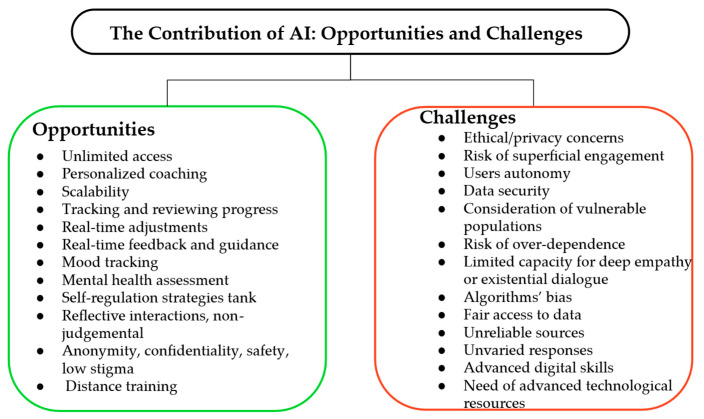
A summary of the main advantages and the challenges derived from the use of AI tools.

Positive and existential psychiatry delivered via AI introduced several ethical considerations. Initially, data privacy and confidentiality remain a concern, especially in sensitive emotional dialogues. Algorithmic bias may influence the quality of support across demographic groups. Overdependence on AI agents may impact autonomy in emotionally vulnerable users. It is important to mention that none of the studies reported major adverse events, but most excluded participants with severe symptoms, reflecting cautious use in clinical practice [74,75]. Figure 7 summarizes the aforementioned opportunities and challenges derived from the use of AI-based technologies.

### 5.3. Limitations of the Review and Included Studies

Several limitations should be acknowledged when interpreting the findings, including the heterogeneity across the included randomized controlled trials. More specifically, the studies differed in intervention type, AI modality, study populations, intervention duration and outcome assessment methods. This heterogeneity primarily reflects the limited availability of closely comparable randomized studies within the emerging field of AI-assisted mental health interventions. The current review aimed to capture the breadth of emerging AI-assisted interventions aligned with positive and existential psychiatry principles. Restricting inclusion to highly similar interventions would have resulted in the exclusion of relevant studies and substantially limited the capacity to address the objectives of the review. Therefore, narrative synthesis was considered the most appropriate methodological approach for integrating findings across the currently heterogeneous literature. Consequently, conclusions regarding overall effectiveness should be interpreted with caution.

It is also important to mention that the included studies did not measure metacompetencies as predefined constructs. The synthesis relied on the conceptual mapping between the reported psychological outcomes and theoretically defined metacompetency domains. This approach was adopted to allow synthesis consistent with the objectives of the review while reflecting the current structure of the available literature, in which integrated measurement of both health outcomes and metacompetencies remains relatively uncommon.

Variability in outcome measures across studies limited cross-trial comparability. Different scales were used to assess anxiety, depression, well-being, or psychological functioning, preventing standardized comparison of effect magnitude across interventions.

The outcomes in several studies were assessed using self-report instruments. While self-report measures are widely used and validated within psychological research, they may be influenced by response bias, expectancy effects, or levels of participant engagement with digital platforms. The aforementioned limitation should therefore be considered when interpreting the reported effects.

Several studies involved relatively small sample sizes and short intervention or follow-up periods. As a result, evidence regarding long-term sustainability of observed mental and emotional health improvements remains limited.

The rapid evolution of artificial intelligence technologies represents an additional limitation. Several included studies were conducted prior to the widespread introduction of contemporary generative AI systems. Although these interventions employed adaptive or automated digital components consistent with AI-supported mental health care, technological capabilities continue to evolve rapidly. Therefore, current findings may not fully reflect the potential or limitations of newer AI architectures.

### 5.4. Implications and Future Directions

**Figure 8:**
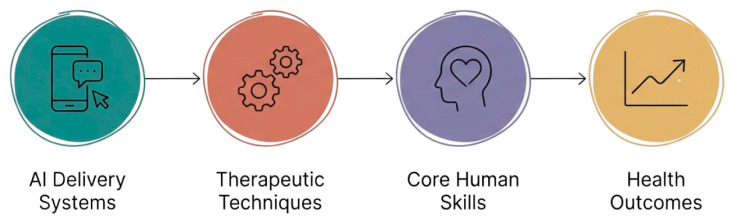
The current systematic review showed that AI is not just a tool: It is a delivery system for proven therapeutic strategies, leading to measurable improvements in well-being.

The evidence demonstrated that AI tools can serve as effective assistive tools to deliver positive psychiatry techniques, especially for young adults [27,60], individuals with mild to moderate anxiety or depression, and populations with limited mental health access [55]. The current study highlights the potential of AI to work in synergy with therapeutic interventions to improve mental and emotional health (Figure 8).

AI-based positive psychiatry interventions may be particularly effective for preventive mental health, early intervention, and well-being enhancement within schools, workplaces, and community health programs. The findings of the current systematic review can be used for designing future research to further explore the link between AI, metacompetency acquisition, and mental health. It would be useful to explore how conversational dynamics promote self-awareness or emotional insight. In addition, future research could provide data from larger RCTs, which may include long-term follow-up to assess maintenance of metacompetency gains. As regards the use of artificial intelligence, future studies can focus on improving affect-recognition accuracy, personalization, and safety constraints.

## 6. Conclusions

This systematic review provided evidence that AI-assisted interventions grounded in positive and existential psychiatry have the potential to provide a proactive healthcare approach for sustainable mental and emotional health. In response to RQ1, the synthesis of twenty-four randomized controlled trials demonstrated that these interventions lead to significant improvements in mental and emotional health outcomes, reducing, for instance, depression, anxiety, and loneliness [52,63]. Regarding RQ2, the review identified that the clinical improvements are associated with the cultivation of self-regulatory metacompetencies such as emotional regulation and self-reflection. Within this healthcare approach, improvements in mood and symptom reduction are viewed as expressions of strengthened metacompetencies.

Although methodological limitations persist, the growing consistency across trials suggests that AI holds substantial promise as a scalable, accessible, and engaging mode of psychological support. The present review contributes to the field by synthesizing empirical evidence through a metacompetency lens, demonstrating that AI-supported interventions may foster deeper psychological mechanisms rather than only reducing symptoms.

These findings imply that integrating AI into positive and existential psychiatry could offer an accessible, scalable approach for strengthening core psychological capacities in both clinical and non-clinical populations. However, large trials with transparent reporting and standardized metacompetency measures are needed. With advancements in generative models and responsible clinical integration, AI systems may become central tools in positive and existential psychiatry interventions.

## Figures and Tables

**Table 1:** Conceptual components underlying AI-assisted positive and existential psychiatry interventions. The table summarizes the theoretical domains informing the framework of the present review, including principles from positive psychiatry and existential psychiatry, the concept of metacompetencies, and the role of artificial intelligence in mental health interventions [7,8,9,10,11,12,13,14,16,17,18,19,20,21,22,23,24,25,26,27,28,29,30].

Conceptual Domain	Core Theoretical Principles	Relevance for AI-Assisted Mental Health Interventions
Positive Psychiatry	Emphasizes psychological strengths, well-being, resilience, optimism, and positive emotional functioning.	AI-based interventions may incorporate strengths-based exercises, gratitude practices, and positive cognitive reframing to support well-being and adaptive coping.
Existential Psychiatry	Focuses on meaning-making, personal responsibility, authenticity, and existential reflection in the context of human suffering and life challenges.	AI conversational agents and reflective tools may facilitate guided reflection, value clarification, and meaning-oriented dialogue supporting existential coping processes.
Metacompetencies	Higher-order psychological capacities enabling individuals to consciously regulate thoughts, emotions, and behaviors across different contexts (e.g., emotional awareness, reflective functioning, resilience, and self-regulation).	AI-supported therapeutic activities may indirectly promote the development of metacompetencies through structured self-reflection, emotional monitoring, and cognitive restructuring exercises.
Artificial Intelligence in Mental Health Care	Use of conversational agents, chatbots, and AI-guided digital platforms to deliver psychological support and behavioral interventions.	AI technologies provide scalable and accessible delivery of therapeutic content and structured mental health support.
Mental and Emotional Health Outcomes	Psychological outcomes commonly assessed in intervention studies include depression, anxiety, stress, well-being, and life satisfaction.	Improvements in these outcomes may occur through enhanced coping capacities and strengthened metacompetencies facilitated by AI-assisted interventions.

**Table 2:** The inclusion and exclusion criteria according to which studies were selected.

Inclusion	Exclusion
(a) Randomized controlled trials (b) Published after 2015 (c) Positive and/or existential psychiatry intervention as the primary type of intervention with the support of AI technologies (d) Studies that assessed mental and emotional health outcomes (e) Studies that employed psychological measurement tools that are associated with mental and emotional health metacompetencies (f) Healthy participants and participants with health-related problems	(a) Systematic reviews, meta-analyses, and book chapters (b) Protocols and design frameworks (c) Studies that did not employ AI design (d) Studies that did not employ positive and existential psychiatry techniques (e) Studies published in languages other than English

**Table 3:** The search strings include the main keywords.

The Search Strings with the Main Keywords
“Positive psychiatry” OR “existential psychiatry” OR “Socratic questioning” OR “gratitude” OR “savoring” OR “cognitive restructuring” OR “mindfulness” OR “affirmation techniques” OR “meaning and purpose-oriented techniques” OR “strength-based techniques” OR “cognitive restructuring” OR “positive reappraisal” AND “Artificial intelligence” OR “conversational agents” OR “generative AI” OR “large language model” OR “virtual coaches”, “intelligent agents” OR “embodied agent” AND “Metacognitive skills” OR “emotional intelligence skills” OR “self-regulation” OR “emotional regulation” OR “self-awareness” OR “emotional awareness” OR “emotional recognition” OR “impulse control” OR “attentional regulation” OR “flexibility” OR “stress management”
AND “Mental health” OR “emotional health” OR “resilience” or “psychological well-being” OR “depression” OR “anxiety” OR “Attention-Deficit/Hyperactivity Disorder” OR “Post-Traumatic Stress Disorder” OR “burnout” OR “Autism spectrum disorder” OR “loneliness”

**Table 4:** A synthesized overview of the randomized controlled trials included in the review, grouped by AI modality.

AI Modality	Number of Studies	Typical Sample Size	Intervention Duration	Main Metacompetencies	Main Outcomes
Conversational AI chatbots	10	30–300	2–8 weeks	Emotional regulation, self-awareness	Reduced depression and anxiety
Generative AI chatbots	3	100–250	4–6 weeks	Cognitive reappraisal, emotional awareness	Reduced depression
Rule-based therapeutic chatbots	4	80–300	10 days–4 weeks	Emotional regulation	Reduced distress
AI-augmented journaling systems	2	50–100	2–4 weeks	Self-reflection, mood regulation	Reduced anxiety
Embodied conversational agents/avatars	3	40–200	3–8 weeks	Emotional awareness	Improved well-being
Social or humanoid AI robots	2	30–120	4–12 weeks	Social awareness	Reduced loneliness

## Data Availability

No new data were created or analyzed in this study. Data sharing is not applicable to this article.

## Supplementary Materials

The following supporting information can be downloaded at: https://www.mdpi.com/article/10.3390/healthcare14060783/s1, Table S1. Search string used in PubMed. Medical Subject Headings (MeSH) and free-text terms were combined using Boolean operators. Table S2. Search string used in Scopus. Searches were conducted using the TITLE-ABS-KEY field. Table S3. Search strategy used in Web of Science Core Collection using the Topic (TS) field. Table S4. Search string used in PsycINFO using controlled vocabulary and keyword searches. Table S5. Search strategy used in Google Scholar. Due to search engine limitations, simplified keyword combinations were applied.

## Appendix A

**Table A1:** Summary table of the AI-based positive and existential psychiatry interventions for mental and emotional health and the role of metacompetency development.

Reference	Country	Positive Existential Intervention Strategy	AI Tool	Sample	Clinical Condition	Duration	Measurements	Study’s Design	Metacompetencies	Main Health Outcomes
Zulfikar et al., 2025 [66]	USA	Savoring positive experiences	AI-augmented journaling	*n* = 55, (*n*_exp_ = 28, *n*_clt_ = 27)	HS	2 weeks	PHQ8, Daily Affect Score	RCT	Self-awareness, mood regulation, memory retention, stress management, and critical thinking	Reduction in anxiety and depression levels
Heinz et al., 2025 [58]	USA	Socratic questioning, reflective listening, and guided exercises	Generative AI chatbot	*n* = 210, (*n*_exp_ = 106, *n*_clt_ = 104), F = 125, M = 78, NB = 6, Μ_age_ = 33.8	ANX, DEP, ED	4 weeks	PHQ-9, GAD-7, WCS, SWED, WAI-SR	RCT	Emotional Awareness, cognitive reframing, values clarification, self-compassion	Reduction in symptoms of depression
Tong et al., 2025 [64]	China	Behavioral activation	Rule-based, topic-specific chatbots	*n* = 285, (*n*_exp_ = 140, *n*_clt_ = 145), F = 216, M = 68, Μ_age_ = 26.45	HS	10 days	SUPPH, eTAP, SCBI, MHLS, PHQ-9, GAD-7	RCT	Emotional awareness and regulation, self-observation	Reduction in psychological distress
Akdogan et al., 2025 [61]	Turkey	Empathetic and reflective dialogues	AI-Chatbot	*n* = 32, (*n*_exp_ = 16, *n*_clt_ = 16), F = 20, M = 12, 18–22 years old	CN, ANX, DEP	3 weeks	HADS	RCT	Emotional self-regulation, self-efficacy, and metacognitive awareness	Lower anxiety and depression
Hung et al., 2025 [65]	Singapore	Gratitude, affirmation techniques, self-reflection, and insight, cognitive reappraisal	AI-Chatbot	Study 1: *n* = 106, Study 2: *n* = 50 participants, 86.8% Female, Μ_age_ = 20.67	HS	Single 10 min session	ESAT,	RCT	Emotional Regulation	Life satisfaction
Indrayanti et al., 2025 [57]	Indonesia	empathetic conversations, hope-inspiring messages	AI-chatbot	*n* = 150, (*n*_exp_ = 80, *n*_clt_ = 80), F = 80, M = 70, Μ_age_ = 64		Single 30 min session	UCLA-LS, WEMWBS, LVS,	RCT	Emotional awareness and regulation	Significant reduction in loneliness, improved psychological well-being and emotional relief
Jiang et al., 2025 [62]	USA	Meditation, motivational messages, guided self-help techniques, and coping strategies	AI-based-chatbot	*n* = 65, (*n*_exp_ = 32, *n*_clt_ = 33), F = 65, M_age_:15.24	DEP, ANX	2 months (3 times per week, 20 min per session)	PCS, GHQ-28	RCT	Self-reflection, cognitive restructuring, and emotional regulation,	Reductions in depression, anxiety, and negative emotional states Reduced psychological distress
Karkosz et al., 2024 [53]	Poland	Socratic questioning	AI chatbot	*n* = 81, (*n*_exp_ = 40, *n*_clt_ = 41), F = 58, M = 21, NB = 2 M_age_ = 26.6	ANX, DEP	2 weeks	CESD-R, PHQ-9, PSWQ, STAI, PANAS, SWLS, R-UCLA	RCT	Cognitive restructuring, emotional regulation, self-awareness, behavioral activation	Depressive and anxiety symptoms’ reduction, decline in loneliness
Kleinau et al., 2024 [55]	Malawi	cognitive restructuring, behavioral activation, gratitude, breathing, relaxation, and meditation techniques (breathing, relaxation, and meditation)	AI chatbot	*n* = 511, (*n*_exp_ = 296, *n*_clt_ = 215), F = 511, age: under 30 years old	HS	8 weeks	GAD-7, PHQ-9, OLBI, ULCA, RS-14	RCT	Self-reflection, self-awareness, stress management, and emotional regulation	Reduced depression, anxiety, and burnout, increased resilience
Robinson et al., 2023 [56]	Australia	Mindfulness techniques	Autonomous humanoid social robot	*n* = 230, (*n*_exp_ = 106, *n*_clt_ = 124), F = 108, M = 122, M_age_ = 29	HS	Single session	RUI, RIS,	RCT	Mindfulness skills	Increased positive feelings. Increased self-satisfaction
Lee et al., 2023 [54]	Germany	Gratitude techniques, self-reflection techniques	AI chatbot	*n* = 133, (*n*_exp_ = 62, *n*_clt_ = 71), F = 84, M = 45, NB: 3, undisclosed: 1, M_age_ = 33.71	HS	3 days	GQ-6, SPANE	RCT	Self-reflection, positive self-expression, recognition skills	Emotional well-being, increased positive emotions, decreased negative emotions, increased gratitude
He et al., 2022 [63]	China	Cognitive restructuring techniques, behavioral activation, and thought recording techniques	AΙ chatbot	*n* = 148, (*n*_exp_ = 49, *n*_clt1_ = 49, *n*_clt2_ = 50), F = 55, M = 93, M_age_ = 18.78	DEP	1 week	PHQ-9, WAQ	RCT	Cognitive-behavioral metacompetencies, emotional regulation, self-compassion, problem solving, and coping skills	Reduction in depressive symptoms, improved mental wellness
Klos et al., 2021 [60]	Argentina	Relaxation techniques, solution-focused strategies, and emotion-focused strategies	AI chatbot	*n* = 181, (*n*_exp_ = 99, *n*_clt_ = 82), F= 158, M = 23, age: 18–33	ANX, DEP	8 weeks	PHQ-9, GAD-7	RCT	Self-reflection, emotional regulation, behavioral activation	Improved emotional well-being, reduced anxiety, and depression
Gardiner et al. 2017 [67]	USA	MBSR	Embodied conversational AI agent	*n* = 61, (*n*_exp_ = 31, *n*_clt_ = 30), F = 61, M_age_ = 35	HS	4 weeks	PSS-4, SEE, The system’s data	RCT	Self-regulation of addictive behaviors, adaptive coping skills, and stress management	Reductions in stress and depressive symptoms, and an increase in mindfulness
Ly et al., 2017 [59]	Sweden	Gratitude, kindness, and reflective practices	AI-based chatbot	*n* = 28, (*n*_exp_ = 14, *n*_clt_ = 14), F = 15, M = 13 M_age_ = 21.1	HS	2 weeks	FS, SWLS, PSS-10	RCT	Self-reflection, self-awareness	Reductions in depression and anxiety symptoms
Fulmer et al., 2018 [52]	USA	Cognitive restructuring, gratitude exercises, emotion-focused coping, mindfulness, and behavioral activation	AI chatbot	*n* = 626, (*n*_exp_ = 208, *n*_clt1_ = 209, *n*_clt2_ = 209), F = 524, M = 102, M_age_ = 24.5	ANX/DEP	8 weeks	PHQ-9, GAD-7	RCT	Self-reflection, emotional awareness, resilience, adaptation, cognitive reframing, self-compassion, empathy	Reduced depression and anxiety, increased emotional well-being
Anan et al. 2021 [46]	Japan	Acceptance and mindfulness-based intervention	AI-assisted smartphone chatting app	*n* = 94 (*n*_exp_ = 48, *n*_clt_ = 46), F = 22, Μ = 72, M_age_ = 41.8	HS	12 weeks	Linear regression analysis	RCT	Management of intense feelings	Improvements in depression and anxiety symptoms
Sturgill et al. 2021 [44]	USA	Positive affirmations, gratitude exercises	AΙ-powered emotional intelligence and mindfulness app	*n* = 99 (*n*_exp_ = 50, *n*_ctl_ = 49), M_age_ = 19.9, F = 27, M = 69	HS	14 weeks, M_eng_=1424 min, 2 sessions per week	GAD-7, PHQ-9, TWI	RCT	Emotional awareness, emotional wellness, stress, and mood regulation	Reduced anxiety, depression
Marcuzzi et al. 2023 [34]	Norway	Mindfulness audio files	AI-based smartphone app	*n* = 294 (*n*_exp_ = 99, *n*_clt1_ = 98, *n*_clt2_ = 97), M_age_ = 50.6, F = 173, M = 121	HS	12 weeks	MSK-HQ, RMQ, PSEQ, B-IPQ	RCT	Self-management of behavior	Improved mental and physical health
Fitzpatrick et al., 2017 [27]	USA	Positive CBT, self-reflection techniques	AI conversational agent	*n* = 70, (*n*_exp_ = 34, *n*_clt_ = 36), M = 23, F = 47, M_age_ = 22.2	DEP, ANX	2 weeks (up to 20 sessions)	PHQ-9, GAD-7, PANAS	RCT	Self-motivation, engagement, and mood regulation	Reduced symptoms of depression and anxiety
Burton et al., 2016 [45]	Romania, Spain, and the UK	Positive CBT, positive reframing, behavioral activation, positive visualizations	Embodied virtual agent	*n* = 27, (*n*_exp_ = 13, *n*_clt_ = 14), M = 9, F = 18, M_age_ = 35.3	DEP	10 sessions	BDI-2, DAS-SF, EQ-5D, QIDS-SR,	RCT	Mood regulation, decreased symptoms of sadness, pessimism, worthlessness, and self-criticism	Improved depression symptoms and mood
Oh et al., 2020 [28]	Korea	Positive CBT	Mobile app-based chatbot	*n* = 41, (*n*_exp_ = 21, *n*_clt_ = 20), M = 20, F = 21, M_age_ = 37.6	PD	4 weeks	PDSS, APPQ, HADS, BSQ, ACQ	RCT	Self-management skills, emotional control, rational thinking, and decreased social phobia	Reductions in panic and anxiety symptoms
Greer et al., 2019 [29]	USA	Gratitude, kindness, savoring, and positive reappraisal	Vivibot chatbot	*n* = 45 (*n*_exp_ = 25, *n*_clt_ = 20), F = 36, Μ = 9, M_age_ = 25	ANX, DEP	4 weeks, 12 sessions	PROMIS, DES	RCT	Positive thinking, positive reappraisal, and stress management	Reduced anxiety and depression symptoms
Jang et al., 2021 [30]	Korea	Behavioral change, mindfulness, and emotional control techniques	Mobile AI-based chatbot	*n* = 46 (*n*_exp_ = 23, *n*_clt_ = 23), F = 26, Μ = 20, M_age_ = 25.1	ADHD	4 weeks	CAARS, QIDS-SR, SAS, PSS	RCT	Attention regulation, self-concept, emotional stability, and self-esteem	Significant reductions in ADHD symptoms, depression, and anxiety

*n*: number of participants, *n*_exp_: experimental group, *n*_ctl_: control group, M_age_: mean age, F: female, M: male, HS: healthy subjects, RCT: Randomized Controlled Trial, FS: Flourishing Scale, PSS-10: The Perceived Stress Scale, CESD-R: Center for Epidemiologic Studies Depression Scale Revised, R-UCLA: UCLA Loneliness Scale, PANAS: Positive and Negative Affect Scale, PHQ-9: Patient Health Questionnaire-9, PSWQ: Penn State Worry Questionnaire, STAI: State-Trait Anxiety Inventory, SWLS: Satisfaction With Life Scale, PCS: Psychological Complaints Scale, GHQ-28: General Health Questionnaire-28, OLBI: Oldenburg Burnout Inventory, RS–14: 14-item Resilience Scale, RUI: Robot Usage Intention, RIS: Robot Incentives Scale, WAQ: Working Alliance Questionnaire, GQ-6: Gratitude Questionnaire—Six Item Form, SPANE: Scale of Positive and Negative Experience, HADS: Hospital Anxiety and Depression Scale, ED: Eating Disorder, GAD-Q-IV: Generalized Anxiety Disorder Questionnaire-IV, WCS: Weight Concerns Scale, SWED: Stanford-Washington University Eating Disorder, WAI-SR: Working Alliance Inventory—Short Revised, SUPPH: Strategies Used by People to Promote Health, eTAP: e-Therapy Attitude and Process Questionnaire, SCBI: Self-Care Behavior Inventory, MHLS: Self-constructed Mental Health Literacy Scale, UCLA LS: Loneliness Scale, WEMWBS: Warwick-Edinburgh Mental Well-Being Scale, LVS: Loneliness Vulnerability Scale, ESAT: emotional state assessment tool.
