# Supplementary material for: Systematic Review of Artificial Intelligence in Positive and Existential Psychiatry: Advancing Mental and Emotional Health Through Metacompetency Development
Source: Healthcare (Basel). 2026 Mar 19;14(6):783. doi: 10.3390/healthcare14060783 (PMC13026824; doi:10.3390/healthcare14060783)
Supplement: Supplementary file 1 [file healthcare-14-00783-s001.zip › SEARCH STRINGS.pdf]

Database-specific search strings used in the systematic review

Table S1. Search string used in PubMed. Medical Subject Headings (MeSH) and free-text terms were combined using Boolean operators.

Concept	Search String
AI technologies	("Artificial Intelligence"[Mesh] OR "artificial intelligence"[tiab] OR AI[tiab] OR chatbot*[tiab] OR "conversational agent*"[tiab] OR "virtual agent*"[tiab] OR "embodied agent*"[tiab] OR robot*[tiab] OR "generative AI"[tiab] OR "large language model*"[tiab] OR "intelligent agent*"[tiab] OR "virtual coach*"[tiab])
Positive/existential psychiatry interventions	("positive psychiatry"[tiab] OR "existential psychiatry"[tiab] OR mindfulness[Mesh] OR mindfulness[tiab] OR gratitude[tiab] OR savoring[tiab] OR "cognitive restructuring"[tiab] OR "positive reappraisal"[tiab] OR "affirmation technique*"[tiab] OR "strength-based"[tiab] OR "meaning in life"[tiab] OR "Socratic questioning"[tiab])
Metacompetencies	("metacognitive skill*"[tiab] OR "emotional intelligence"[tiab] OR "self-regulation"[tiab] OR "emotional regulation"[tiab] OR "self-awareness"[tiab] OR "emotional awareness"[tiab] OR "emotional recognition"[tiab] OR "impulse control"[tiab] OR "attentional regulation"[tiab] OR flexibility[tiab] OR "stress management"[tiab])
Mental health outcomes	("mental health"[Mesh] OR "mental health"[tiab] OR "emotional health"[tiab] OR resilience[tiab] OR "psychological well-being"[tiab] OR depression[Mesh] OR depression[tiab] OR anxiety[Mesh] OR

	anxiety[tiab] OR burnout[tiab] OR loneliness[tiab] OR "post-traumatic stress disorder"[tiab] OR ADHD[tiab] OR "autism spectrum disorder"[tiab])
Final combined search	(AI technologies) AND (Positive/existential psychiatry interventions) AND (Metacompetencies) AND (Mental health outcomes)
Filters	English, publication year ≥2015

Table S2. Search string used in Scopus. Searches were conducted using the TITLE-ABS-KEY field.

Search
TITLE-ABS-KEY(("artificial intelligence" OR AI OR chatbot* OR "conversational agent*" OR "virtual agent*" OR "embodied agent*" OR robot* OR "generative AI" OR "large language model*" OR "intelligent agent*" OR "virtual coach*") AND ("positive psychiatry" OR "existential psychiatry" OR mindfulness OR gratitude OR savoring OR "cognitive restructuring" OR "positive reappraisal" OR "affirmation techniques" OR "strength-based" OR "meaning in life" OR "Socratic questioning") AND ("metacognitive skill*" OR "emotional intelligence" OR "self-regulation" OR "emotional regulation" OR "self-awareness" OR "emotional awareness" OR "emotional recognition" OR "impulse control" OR "attentional regulation" OR flexibility OR "stress management") AND ("mental health" OR "emotional health" OR resilience OR "psychological well-being" OR depression OR anxiety OR burnout OR loneliness OR "post-traumatic stress disorder" OR ADHD OR "autism spectrum disorder")) AND PUBYEAR > 2014 AND (LIMIT-TO(LANGUAGE, "English"))

Table S3. Search strategy used in Web of Science Core Collection using the Topic (TS) field.

TS=(("artificial intelligence" OR AI OR chatbot* OR "conversational agent*" OR "virtual agent*" OR "embodied agent*" OR robot* OR "generative AI" OR "large language model*" OR "intelligent agent*" OR "virtual coach*") AND ("positive psychiatry" OR "existential psychiatry" OR mindfulness OR gratitude OR savoring OR "cognitive restructuring" OR "positive reappraisal" OR "affirmation techniques" OR "strength-based" OR "meaning in life" OR "Socratic questioning") AND ("metacognitive skill*" OR "emotional intelligence" OR "self-regulation" OR "emotional regulation" OR "self-

awareness" OR "emotional awareness" OR "emotional recognition" OR "impulse control" OR "attentional regulation" OR flexibility OR "stress management") AND ("mental health" OR "emotional health" OR resilience OR "psychological well-being" OR depression OR anxiety OR burnout OR loneliness OR "post-traumatic stress disorder" OR ADHD OR "autism spectrum disorder")) Refined by: Language = English; Timespan = 2015–2025

Table S4. Search string used in PsycINFO using controlled vocabulary and keyword searches.

Search
(DE "Artificial Intelligence" OR "artificial intelligence" OR AI OR chatbot* OR "conversational agent*" OR "virtual agent*" OR "embodied agent*" OR robot* OR "generative AI" OR "large language model*" OR "virtual coach*") AND ("positive psychiatry" OR "existential psychiatry" OR mindfulness OR gratitude OR savoring OR "cognitive restructuring" OR "positive reappraisal" OR "affirmation techniques" OR "strength-based" OR "meaning in life" OR "Socratic questioning") AND ("metacognitive skill*" OR "emotional intelligence" OR "self-regulation" OR "emotional regulation" OR "self-awareness" OR "emotional awareness" OR "emotional recognition" OR "impulse control" OR "attentional regulation" OR flexibility OR "stress management") AND ("mental health" OR "emotional health" OR resilience OR "psychological well-being" OR depression OR anxiety OR burnout OR loneliness OR "post-traumatic stress disorder") Filters: English; 2015–2025

Table S5. Search strategy used in Google Scholar. Due to search engine limitations, simplified keyword combinations were applied.

Search
("artificial intelligence" OR chatbot OR "conversational agent" OR "generative AI") AND ("positive psychiatry" OR "existential psychiatry" OR mindfulness OR gratitude OR "cognitive restructuring") AND ("emotional regulation" OR "self-awareness" OR "emotional intelligence") AND ("mental health" OR wellbeing OR depression OR anxiety)
Filters applied: publication year ≥2015, English language, relevance ranking.
